# Suicide Prevention in Older Adults: Evidence-Based Approaches for Care

**DOI:** 10.1093/geroni/igaa057.2129

**Published:** 2020-12-16

**Authors:** Luming Li

## Abstract

Suicide in older adults is a major public health concern. Data of suicide rates of older adults from the Centers for Disease Control suggests that suicide is more frequent in older adults and warrants further examination of treatment and public health prevention approaches. Risk factors for suicide in the elderly include functional disability, multiple chronic physical conditions, and social isolation. Several advances have been made in healthcare policy to address practical, evidence-based approaches to preventing suicide and treating behavioral health conditions such as depression, including collaborative care and the Zero Suicide model. This symposium will focus on reviewing the epidemiology and evidence-based approaches for suicide prevention and mental health treatment for older adults. In this presentation, the presenters describe the current trends in suicide rate in older adults in the United States, indicate risk factors (both modifiable and non-modifiable), and present about the collaborative care and Zero Suicide models. Speakers will emphasize the role of these two models in suicide prevention and population-based behavioral healthcare. The presenters will also highlight examples of policy changes and provide recommendations for regulators and hospital systems to adopt these evidence-based models of care for caring for older adults at risk for suicide.

